# A Hardware Implementation of SNN-Based Spatio-Temporal Memory Model

**DOI:** 10.3389/fnins.2019.00835

**Published:** 2019-08-09

**Authors:** Kefei Liu, Xiaoxin Cui, Yi Zhong, Yisong Kuang, Yuan Wang, Huajin Tang, Ru Huang

**Affiliations:** ^1^Institute of Microelectronics, Peking University, Beijing, China; ^2^National Key Laboratory of Science and Technology on Micro/Nano Fabrication, Peking University, Beijing, China; ^3^College of Computer Science, Sichuan University, Chengdu, China

**Keywords:** memory model, brain-inspired, spike neural network, spatio-temporal memory, neuromorphic hardware, FPGA

## Abstract

Simulating human brain with hardware has been an attractive project for many years, since memory is one of the fundamental functions of our brains. Several memory models have been proposed up to now in order to unveil how the memory is organized in the brain. In this paper, we adopt spatio-temporal memory (STM) model, in which both associative memory and episodic memory are analyzed and emulated, as the reference of our hardware network architecture. Furthermore, some reasonable adaptations are carried out for the hardware implementation. We finally implement this memory model on FPGA, and additional experiments are performed to fine tune the parameters of our network deployed on FPGA.

## 1. Introduction

**Figure 1:**
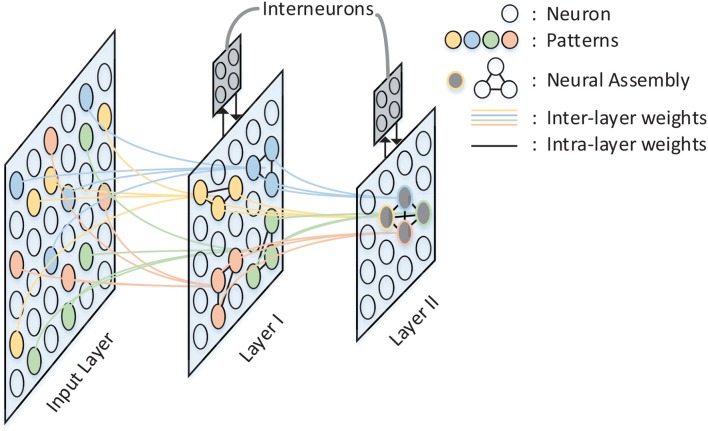
The architecture of the STM model. There are three layers in the network: Input Layer, Layer I, and Layer II. Inter-layer connections between layers are illustrated by colored lines, while intra-layer connections are black lines and exist only within Layer I and Layer II. Neurons with the same color in each layer form a neural assembly and denote the same memory item.

A spatio-temporal memory model has been proposed by Hu et al. (2016). In this model, information is hierarchically stored in a structured spiking neural network as shown in Figure 1. Compared with pre-existing memory models, the major contribution of this model lies in the memory formulation implemented by temporal population coding and temporal learning. Each pattern stored in the network is represented by tens of neurons, with the information being encoded by the precise time of spikes. During the learning phase, the inter-layer synaptic weights are updated in accordance with the tempotron learning rule (Gütig and Sompolinsky, 2006), while intra-layer synaptic plasticity is modified by spike timing dependent plasticity (STDP) (Caporale and Dan, 2008). Throughout the recall phase, hetero-associated memory is stored in the connections between input layer and layer I, which enables a timely response for a particular neuron assembly in layer I whenever a new pattern is introduced to input layer. Auto-associated memory refers to the capability that a subset of neurons from a particular neural assembly arouse the rest of that assembly. Moreover, the capability of pattern completion in this memory model is maintained by the lateral connections within neuron assemblies of layer I. Episodic memory could be considered as an abstraction of temporally consecutive simple memory patterns. A silent neuron assembly in layer II may be triggered by the others by means of inter-assembly connections as long as a pattern sequence has been learned during learning phase. In the recall phase, a pattern is recalled if the neuron assembly related to that pattern fires spikes repetitively. In this case, after-depolarizing potential (ADP) (Jensen et al., 1996) of neurons and theta oscillation (O'Keefe and Recce, 1993) are both important to the repetitive firing of assemblies coding for memory items. The STM model provides a comprehensive substrate to elucidate the complex process of memory formulation and organization in virtue of complex spiking neural dynamics. In this work, we implement the STM model on FPGA.

Some neuromorphic efforts have been made recently to simulate human brain. The SpiNNaker chip, proposed by University of Manchester, integrates 18 processing cores, in which each one could configure ~1,000 biologically-plausible neurons (Painkras et al., 2013). For the TrueNorth chip proposed by IBM in 2015, the chip itself is composed of 4,096 neurosynaptic cores, containing an aggregate of 1 million neurons and 256 million synapses (Akopyan et al., 2015). And it achieves even better performance both in accuracy and power on some classification tasks than other so-called state-of-the-art approaches (Esser et al., 2016). Loihi is another neuromorphic chip proposed by Intel in 2018, comprising 128 neuromorphic cores with fully integrated SNN, where each neuromorphic core implements 1,024 primitive spiking neural units (Davies et al., 2018). Please note that the neuromorphic chips mentioned above are used to run ANN tasks by many researchers. For instance, an object (letters a–z) recognition task containing 8,198 neurons was performed on SpiNNaker as shown in Orchard et al. (2015), and a convolutional neural network for CIFAR 10 classification was deployed on one TrueNorth chip employing 4,042 cores containing 4,042*256 neurons in Esser et al. (2016). However, most of the tasks that our brain deals with everyday are cognitive tasks, such as association and memory search, instead of classifications. Despite ~100 billion neurons and 1,000–10,000 synapses per neuron in our brains, some cognitive tasks do not need so many neurons. As for the STM model, a few hundreds of neurons are sufficient to store and recall several memory items. Hence, we implement the STM model on FPGA in this paper, and we plan to propose our configurable neuromorphic chip recently.

The remaining part of this paper is organized as follows: in section 2, we introduce the adaptations of STM model, to make it more suitable for hardware implementation. In section 3, we describe the hardware implementation details. In section 4, we discuss the impacts of different model parameters on the model behavior and the cost of hardware.

## 2. Materials and Methods

### 2.1. Neuron Model

The spike response model (SRM) (Maass and M Bishop, 1998) is adopted in the original STM network. And the neural dynamics of SRM is described as function (1), where *v*(*t*) is its membrane potential at time *t* representing its state. *t*_*i*_ and *t*_*j*_ denote firing times of the pre-synaptic neuron *i* and the post-synaptic neuron j, respectively. *w*_*ij*_ is the synaptic efficacy from neuron j to neuron i. *A*_*ADP*_, τ_*ADP*_, *V*_*norm*_, τ, *and τ*_*s*_ are all constants and *h*^*ext*^(*t*) is external stimulating input (Hu et al., 2016).

(1) $$\begin{array}{l} v_{i}(t)=A_{ADP}\frac{t-t_{i}}{\tau_{ADP}}exp(1-\frac{t-t_{i}}{\tau_{ADP}})+\sum_{j}w_{ij}V_{norm}(exp(-\frac{t-t_{j}}{\tau })\\ \;-exp(-\frac{t-t_{j}}{\tau_{s}}))+h^{ext}(t) \end{array}$$

Obviously, operations including addition, complex multiplication and exponent arithmetic are needed for SRM. By contrast, only addition is employed by the leaky integrate-and-fire (LIF) (Stein, 1965) neuron model, thus allowing mitigating the hardware cost (multiplier and exponential arithmetic unit) greatly. Therefore, we replace SRM with LIF neuron model in the STM hardware implementation. LIF is one of the most widely used models for analyzing the behavior of neural systems, and the membrane potential of the LIF neuron evolves according to three basic operations:

1. Synaptic Integration.
  (2) $$\begin{array}{l} v_{i}\left(t\right)=v_{i}\left(t-1\right)+\overset{n-1}{\underset{j=0}{\sum }}x_{j}\left(t\right)w_{ij} \end{array}$$
  where *v*_*i*_(*t*) describes the membrane potential of neuron *i* at time *t*, and *w*_*ij*_ denotes the synaptic weight from neuron j to neuron i. *x*_*j*_(*t*) is the input value to neuron i from neuron j, and *x*_*j*_(*t*) ∈ {0, 1} for depicting binary spike neuron networks.
2. Leaky.
  (3) $$\begin{array}{l} v_{i}\left(t\right)=v_{i}\left(t\right)-v_{l} \end{array}$$
  *v*_*l*_ is the leak value of the LIF neuron. The constant is subtracted in each time step and such operation guarantees the recovering mechanism so as to keep neurons available for the next stimulus, when no input spike occurs for long time. The leak value may vary from neuron to neuron in a trained network.
3. Fire and Reset.
  (4) $$\begin{array}{l} if\;v_{i}(t)\geq v_{thr}:\\ \;Fire\;and\;v_{i}(t)=v_{reset}\\ else:\;\\ \;pass \end{array}$$
  Finally, the neuron compares its membrane potential with the threshold *v*_*thr*_. If the membrane potential exceeds the threshold value, the neuron fires a spike and reset its membrane potential to *v*_*reset*_.

### 2.2. After-Depolarizing Potential (ADP) and Theta Oscillation

**Figure 2:**
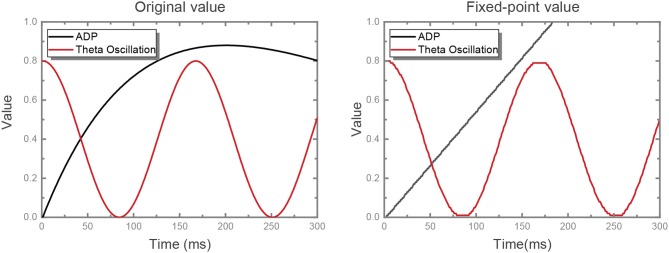
ADP and theta oscillation values. The original ADP kernel is $A_{ADP}\frac{\Delta t}{\tau_{ADP}}exp\left(1-\frac{\Delta t}{\tau_{ADP}}\right)$, non-linearly. We replace it with a linear kernel with a reasonable slope, while the shape of theta oscillation is unchanged. At first we set the slope for the linear kernel as the value at which ADP meets its maximum value at exactly the same time as the original kernel. On this basis, the slope was set as 0.0055 after some experiments. Then, we quantize the value of both ADP and theta oscillation with fixed-point precision.

The after-depolarizing potential of pyramidal cells and theta oscillation in the brain are plugged into the neural dynamics of the original spike response model (SRM). The two properties employed above serve as a mechanism to maintain neuron's status through repetitive firings. However, the LIF neuron model does not support the expected properties on account of complex non-linear functions including exponentiation and multiplication. Therefore, we take both ADP and theta oscillation as external stimulating inputs, and fix-point their values in consideration of hardware cost. The updated membrane potential of neurons with ADP and theta oscillation can be rewritten as:

(5) $$\begin{array}{l} v_{i}\left(t\right)=v_{i}\left(t-1\right)+\Delta ADP\left(t-t_{i}\right)+\Delta \theta \left(t\right) \end{array}$$

where *t*_*i*_ is the last firing timing of neuron i and Δ denotes the amplitude variation between contiguous time steps. We adjust the original ADP dynamics into linear dynamics and fix-point the values of ADP and theta oscillation with a precision of 0.01, where both the original value and the fixed-point value are shown in Figure 2. Some neurons are configured as θ neurons to implement theta oscillation stimulus in connection with other neurons in the network in a manner of fixed synaptic weights, which will be discussed detailedly in the following part. In the meanwhile, the ADP stimulus is handled in the same way.

**Figure 3:**
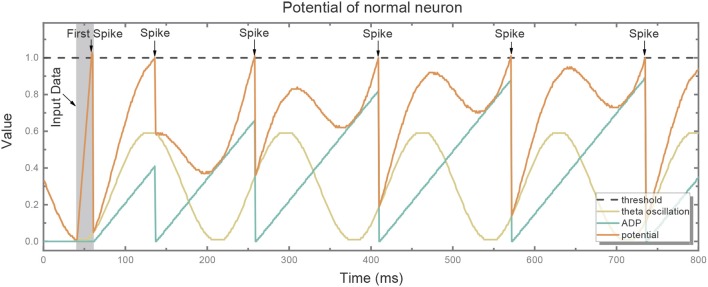
The potential (red line) of the normal neuron after generating the first spike consists of two components: slowly ramping-up ADP (blue line) and theta oscillation (green line). When the ADP meets near-peak theta stimuli, its potential exceeds the threshold (gray dotted line) and it fires again.

Figure 3 depicts the potential trend of a neuron with ADP and theta oscillation after generating the first spike. The potential consists of two components, i.e., the slowly ramping up ADP and theta oscillation. When the ADP meets near-peak theta stimuli, neuron's potential exceeds the threshold and it fires again. Therefore, the information encoded by the neuron is temporally stored in theta cycles.

### 2.3. Fix-Point Processing

**Figure 4:**
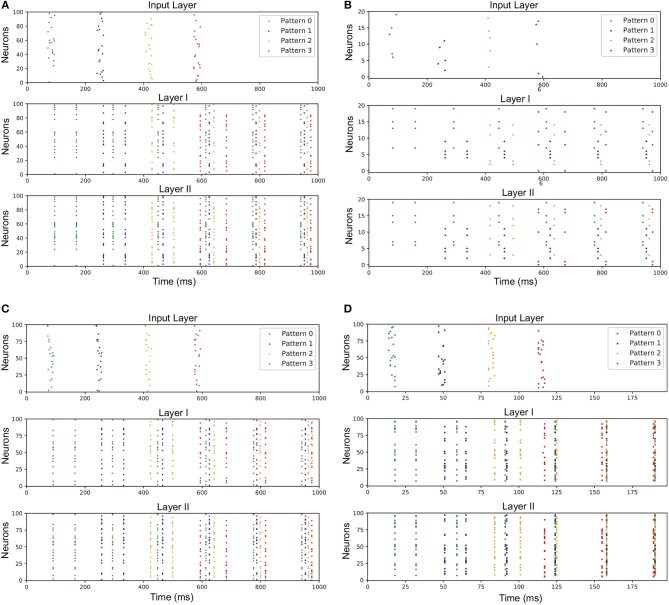
Network performance under various conditions. Spikes of neurons representing for different patterns are marked with different colored dots in the figure. The correct response of layer-I neurons to different input patterns is exactly the hetero-associated memory like recognizing a letter. Then the information is kept in layer I and relevant layer-II neurons are aroused. Therefore, different patterns can compose more complex memory like a word, which is called episodic memory. **(A)** Network trained under modified ADP and theta oscillation and fixed-point parameters. **(B)** Network when input neural assembly size is 5. **(C)** Network when overlap ratio of input neural assembly is 75%. **(D)** Network when gamma period is 5 ms.

To reduce the memory overhead of hardware implementation, we fix-point the weights, leaks and thresholds of the network. During the training phase, the thresholds of all the neurons in the network are set as 1, while the other parameters are all floating-point numbers. When the training is completed, the weights and leaks are rounded to signed 11-bit integers. 11-bit weight exceeds our demand actually. We chose 11-bit weight to express −1024 to 1023, which is not fully used in this implementation (0–300 used), but this gives us more chances to improve our model by fine turning the weights. And 65% memory for weights are reduced after fix-point process. Table 1 gives an overview of the fixed-point values of the previous parameters in a certain case of STM network, in which all the adaptations are applied. One can affirm that the network presents both associated memory and episodic memory correctly as demonstrated in Figure 4A. It should be noted that there is no online learning on the hardware, where both STDP and tempotron are turned off.

**Table 1:** Fixed-point value of parameters.

**Para**	**Layer I**	**Layer II**
Max weight	300	90
Min weight	0	0
Leak	45	28
Threshold	1,000	1,000

## 3. Results

### 3.1. Network Architecture

**Figure 5:**
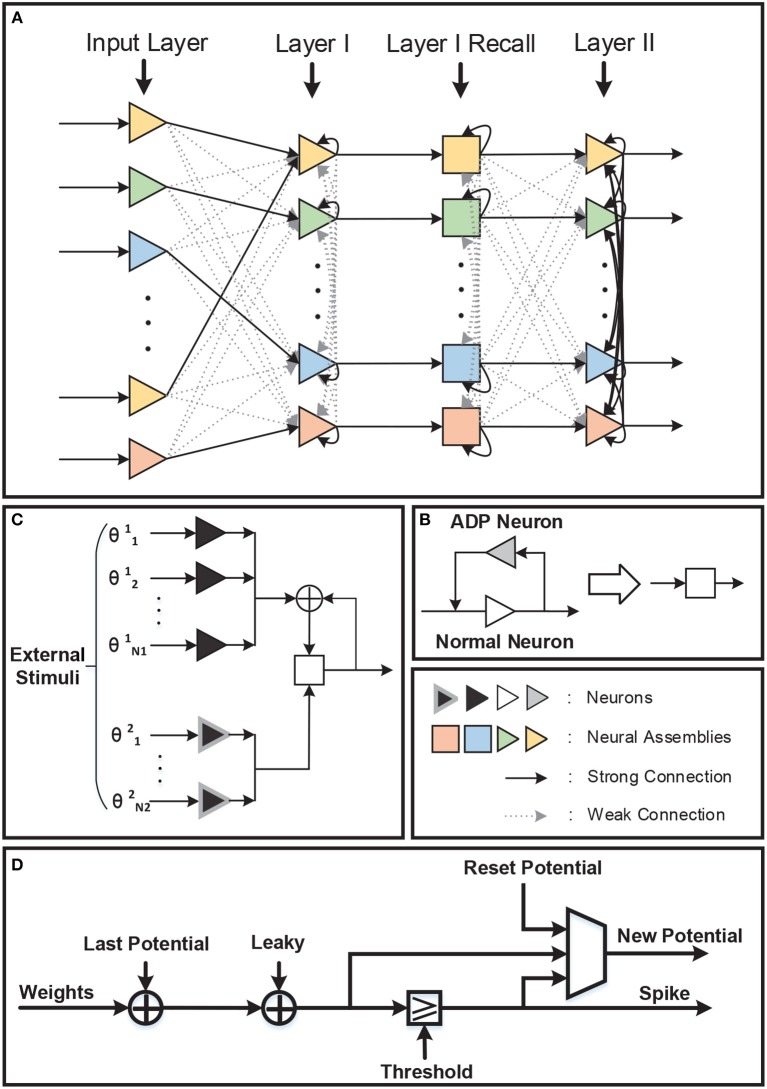
**(A)** There are four layers in the network. One recall layer is needed because we take different methods to achieve repetitive firings from the original model. **(B)** Each neuron in the recall layer is attached to one ADP neuron to realize ramping-up potential after generating spikes. And the ADP neuron will fire continuously once activated. **(C)** Theta oscillation in the recall layer is separated into two parts: the theta oscillation amplitude of the last evaluating period (θ^1^) and the variation of the theta oscillation amplitude between this period and last period (θ^2^). **(D)** Neuron block diagram.

The network architecture is shown in Figure 5A. There are four layers in the network: Input Layer, Layer I, Layer I Recall, and Layer II. Compared with the original STM model network, a recall layer is adopted to achieve repetitive firings. To be specific, the recall layer has the same number of normal LIF neurons as layer I in order to respond to different inputs and transmit stimulus to layer II. Each normal neuron in the recall layer is connected to the corresponding neuron in layer I. What counts is that the after-depolarizing potential (ADP) of normal neuron in the recall layer is realized by the ADP neuron attached to it (Figure 5B). The ADP neuron is also a LIF neuron, with its reset-potential being set the same as its threshold while the leak keeps 0. As a consequence, it shall fire continuously once it is activated unless it receives inhibitory signals. Such process is not displayed in the figure. Acting like a chain of events, the potential of the normal neuron will increase as time goes by owing to the stimuli from the ADP neuron. Besides those mentioned above, normal neurons are also stimulated by θ neurons (inducing the theta oscillation). θ neurons can be categorized into two types: θ^1^ neurons and θ^2^ neurons (Figure 5C). They are fully connected to all the normal neurons in the recall layer and their weights are all ±*w*_θ_. The θ neurons are activated by external stimulus and the number of θ neurons activated in each evaluating period depends on the theta oscillation amplitude. In particular, θ^1^ neurons' stimulus denotes the theta oscillation amplitude of the last evaluating period, while θ^2^ neurons represent the variation of the theta oscillation amplitude between current period and last period. A θ^1^ neurons' stimulus is needed only when normal neuron fires and resets. In this way, the integrated theta oscillation is plugged into normal neurons' potential.

Interneurons in Layer I, Layer I Recall, and Layer II are omitted in Figure 5A for simplicity. Each interneuron should be stimulated by the neural assembly that corresponds to the same pattern it represents for and should provide inhibition to all the other neural assemblies in the same layer. In this way, firings of different neural assemblies are separated into several gamma cycles and become distinguishable temporally.

For hardware implementation, the neural assembly sizes of input layer, layer I and layer II are set to be 10, 8, 10, respectively, and the overlap ratio among input layer neural assemblies is 50%. Above all, 109 neurons are consumed for the network to store four associative memory items and one episodic memory item, including 97 normal neurons and 8 interneurons. Besides, 32 ADP neurons and certain θ neurons are needed in Layer I Recall. The number of θ neurons used depends on the theta period and weight precision. We finally set theta period as 66 ms and the max amplitude as 0.6, thus 60 θ^1^ neurons and 6 θ^2^ neurons are employed and *w*_θ_ is 0.01. The input layer is not included on the hardware, where the input stimuli are routed to layer I neurons directly. As a total, 178 LIF neurons are realized on FPGA.

### 3.2. Neuron Block

The block diagram of the basic unit, LIF neuron, is shown in Figure 5D. In each evaluating period, which is corresponding to real-time millisecond, the neuron reads its parameters from memory, updates its potential by adding up all the valid input weights and executes leak. If its potential exceeds the threshold, the neuron resets its potential and transmits a spike event. If not, the potential keeps unchanged. Then the neuron writes its new potential back to memory.

### 3.3. Hardware Overhead

Each neuron on FPGA is represented by four parameters: 14-bit signed potential, 8-bit signed leak, 14-bit signed reset-potential and 14-bit signed threshold, which sums up to 50 bits memory space. What's more, the 8,512 synapses count most in the network and each synapse stores a 11-bit signed weight. Additional 178 bits are occupied to store the spikes generated in each evaluating period. The final cost of memory is summarized (Table 2).

**Table 2:** Memory cost.

**Item**	**Single cost (b)**	**Number**	**Total cost (kb)**
Neuron	50	178	8.9
Weight	11	8,512	93.632
Spike	1	178	0.178
Total	–	–	102.71

**Figure 6:**
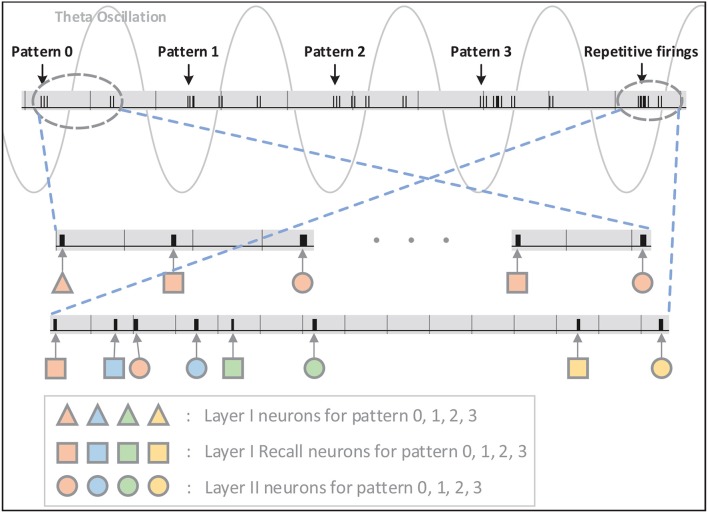
The signal indicating firings of neurons on FPGA is shown in this figure. The firings are illustrated in short black bars. As can be seen, inputs of pattern 0–3 are introduced to the network at valleys of different theta cycles and Layer-I and Layer-II neurons respond selectively and repetitively to the stimulation in the same order. The status of Layer-II neurons is maintained through repetitive firing in the following theta cycles without any inputs.

The FPGA chip used in this realization is Kintex UltraScale FPGA KU115, and the FPGA board we use is custom designed by ourselves. Besides, vivado design suite was applied to deploy the network on the FPGA board. The usage of the FPGA resource is shown in Table 3 and the FPGA result is illustrated in Figure 6, in which the input data is randomly generated for functional verification.

**Table 3:** FPGA resource cost.

**Resource**	**Estimation**	**Available**	**Utilization %**
LUT	5,535	663,360	0.83
FF	4,427	1,326,720	0.33
BRAM	28	2,160	1.30
IO	190	702	27.07
BUFG	1	1,248	0.08

## 4. Discussion

In order to tune appropriate model parameters for hardware implementation, we have performed plenty of simulations to have an insight into the characteristics of STM model. First, we decrease the size of neural assembly in each layer continuously. Second, we increase the overlap of neurons between different neural assemblies. And then, we apply different gamma and theta periods.

### 4.1. Size of Neural Assembly

Population coding is applied in the STM model. Each input item is coded by a specific combination of neurons in each layer. Obviously, the greater the size of neural assembly is, the more hardware resources it consumes. Therefore, it is necessary for us to weigh the tradeoff between the size of neural assembly and the performance of STM network. The neuron number of input neural assembly ranges from 1 to 25 continuously. And the size of layer I neural assembly keeps as three-quarters of that of input layer, while layer II stays the same as input layer.

**Figure 7:**
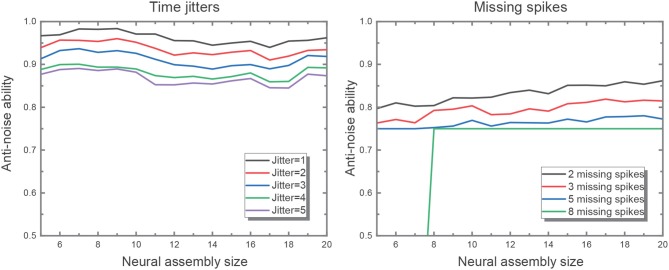
The figure on the left shows the network performance with different time jitters in the input. The performance drops as the time jitter increases from 1 to 5, but it is not sensitive to assembly size. It is evident that the larger input assembly size leads to greater time jitters introduced to the network. And the figure on the right illustrates better network performance in the wake of larger input assembly size when fixing the same missing spike number.

The result under circumstance of 5 for input neural assembly size is shown in Figure 4B, indicating that with the decrease of neural assembly size, the network performance stays almost unaffected. It demonstrates that the size of neural assembly coding for single item is not a principal factor to achieve fundamental functions of STM network. But obviously, the fewer neurons coding for single item, the worse anti-noise property the network may have. Hence, another experiment is conducted to investigate the correlation between neural assembly size and the anti-noise property of STM model. The noise here is expressed as jitter at the moment of individual spikes or missing spikes of input patterns. And the anti-noise property is considered as the capability to generate an output pattern which keeps highly correlated with its corresponding target pattern. The correlation is measured according to (Schreiber et al., 2003):

(6) $$\begin{array}{l} R_{corr}=\frac{\vec{s_{a}}\cdot \vec{s_{d}}}{\left|\vec{s_{a}}||\vec{s_{d}}\right|} \end{array}$$

where *s*_*a*_ and *s*_*d*_ are the filtered actual and desired output spike trains, respectively. The output spike trains are convolved with a Gaussian filter of a given width, σ_*c*_, which is set to be 2 ms. The experiment is divided into two parts. For the first, we shift input spikes by intervals randomly drawn from a Gaussian distribution with mean 0 and variance [1, 5] ms without missing spikes. For the second, we randomly (with uniform distribution) remove 2, 3, 5, 8 spikes from input patterns but leave spike timings unchanged. We did the two parts above with input assembly size decreasing from 20 to 5. And both cases were repeated for 50 times to get the averaged performance. Figure 7 verifies the robustness of the network to time jitter with regard to assembly size. Nevertheless, larger assembly size may ensure higher robustness to missing spikes, which means the network is capable of recalling one pattern even when some features of the inputs are missing. Hereby, we set input neural assembly size as 10 in consideration of both hardware cost and anti-noise capability.

### 4.2. Overlap of Neural Assemblies

Neurons in the same neural assembly encode different features of one specific memory item, hence similar memory items ought to share certain common features. As a result, neural assemblies encoding those items should overlap mutually. Nevertheless, the overlap among neural assemblies may increase the memory capacity of a given network even in the case of the same neuron number.

To figure out the impacts of the overlap degree on the network performance, we generate input patterns with the overlap ratio ranging from 5 to 75% (step length equals to 5%). We find that even though the overlap ratio is increased to 75%, the network still succeeds in learning the target features. The result is drawn in Figure 4C. It indicates that the proposed model is qualified to learn tiny differences between patterns. In our actual hardware implementation, the overlap ratio is set as 50%.

### 4.3. Period of Theta and Gamma Oscillation

In our proposed model, each memory item is encoded by firings in different gamma cycles. Specifically, memorized items representing for episodic memory are serially activated in sequential gamma subcycles of one theta period. It suggests that the recall rate is negatively related to gamma period. Thus accordingly, we expect to attain faster hardware speed by decreasing gamma period. In the experiment, gamma period is set to be 1–25 ms, with theta to gamma frequency ratio being 3/20 to keep the memory capacity unchanged referring to Kamiński et al. (2011). And for the reason that the repetitive firing of neuron assembly is based on the matching of ADP and theta oscillation, the ratio of ADP time constant and gamma period is set as a constant 12 simultaneously for the sake of simplicity.

The experimental results reveal that the gamma period has relatively little impact on the inter-layer connectivity. In spite of that, proper intra-layer connectivity depends heavily on the repetitive firings of neurons at appropriate timing. In the course of decreasing the gamma period, we reveal the fact that the poor matching of after-depolarizing potential (ADP) and external theta input may lead to temporally undistinguishable patterns in Layer II. The result when gamma period is 5 ms is illustrated in Figure 4D.

## 5. Conclusion

In this work, we implement a hierarchical memory model on FPGA. Population coding is employed in this model where information is transmitted as temporal structured spikes. Such mechanism is more preferred in terms of the conformity with our brains. Thus, temporal learning rules, i.e., tempotron and STDP, are applied accordingly. In particular, simple LIF neuron model is adopted as basic elements of the network out of the purpose of hardware-friendly. Moreover, four associative memory items and one episodic memory item are stored in the network and can be recalled properly. For the consideration of hardware cost and memory recall speed, additional validation experiments are performed to tune appropriate network parameters. Studies focusing on deploying deep neuron network on neuromorphic hardware have attracted most of the attention. However, classic recognition and classification tasks are limited to achieve real intelligence, hence this work is a preliminary attempt to build neuromorphic cognitive systems on hardware.

## Data Availability

All datasets analyzed for this study are included in themanuscript and the supplementary files.

## Author Contributions

KL and XC designed the hardware model together. KL performed all of the experiments and wrote this manuscript. YZ and YK helped to implement this model on FPGA. HT gave some suggestions about STM model adaptations. YW and RH assisted in editing.

### Conflict of Interest Statement

The authors declare that the research was conducted in the absence of any commercial or financial relationships that could be construed as a potential conflict of interest.
